# Two Paralogous Gb3/CD77 Synthases in Birds Show Different Preferences for Their Glycoprotein and Glycosphingolipid Substrates

**DOI:** 10.3390/ijms22189761

**Published:** 2021-09-09

**Authors:** Anna Bereznicka, Krzysztof Mikolajczyk, Katarzyna Szymczak-Kulus, Katarzyna Kapczynska, Edyta Majorczyk, Anna Modlinska, Tomasz Piasecki, Radoslaw Kaczmarek, Marcin Czerwinski

**Affiliations:** 1 Laboratory of Glycobiology, Department of Immunochemistry, Hirszfeld Institute of Immunology and Experimental Therapy, Polish Academy of Sciences, 53-114 Wroclaw, Poland; anna.bereznicka@hirszfeld.pl (A.B.); krzysztof.mikolajczyk@hirszfeld.pl (K.M.); katarzyna.szymczak-kulus@hirszfeld.pl (K.S.-K.); radoslaw.kaczmarek@hirszfeld.pl (R.K.); 2 Laboratory of Medical Microbiology, Department of Immunology of Infectious Diseases, Hirszfeld Institute of Immunology and Experimental Therapy, Polish Academy of Sciences, 53-114 Wroclaw, Poland; katarzyna.kapczynska@hirszfeld.pl; 3 Faculty of Physical Education and Physiotherapy, Opole University of Technology, 45-758 Opole, Poland; E.Majorczyk@po.edu.pl; 4 Department of Epizootiology and Clinic of Birds and Exotic Animals, Wroclaw University of Environmental Sciences, 50-366 Wroclaw, Poland; ana.modlinska@gmail.com (A.M.); tomasz.piasecki@upwr.edu.pl (T.P.)

**Keywords:** Gb3/CD77 synthase, glycosyltransferase, Shiga toxin, birds

## Abstract

Most glycosyltransferases show remarkable gross and fine substrate specificity, which is reflected in the old one enzyme-one linkage paradigm. While human Gb3/CD77 synthase is a glycosyltransferase that synthesizes the Galα1→4Gal moiety mainly on glycosphingolipids, its pigeon homolog prefers glycoproteins as acceptors. In this study, we characterized two Gb3/CD77 synthase paralogs found in pigeons (*Columba livia*). We evaluated their specificities in transfected human teratocarcinoma 2102Ep cells by flow cytofluorometry, Western blotting, high-performance thin-layer chromatography, mass spectrometry and metabolic labelling with ^14^C-galactose. We found that the previously described pigeon Gb3/CD77 synthase (called P) can use predominately glycoproteins as acceptors, while its paralog (called M), which we serendipitously discovered while conducting this study, efficiently synthesizes Galα1→4Gal caps on both glycoproteins and glycosphingolipids. These two paralogs may underlie the difference in expression profiles of Galα1→4Gal-terminated glycoconjugates between neoavians and mammals.

## 1. Introduction

Glycosyltransferases (GTs) are a large group of enzymes that catalyze the transfer of a sugar residue from an activated sugar donor to the acceptor molecule forming a glycosidic bond. Glycosyltransferases synthesize carbohydrate chains on glycoproteins, glycosaminoglycans, glycosphingolipids and other glycoconjugates, usually showing high donor and acceptor specificity. They play many roles in immune response, signal transduction and infections [1,2]. Human Gb3/CD77 synthase (UDP Gal:lactosylceramide α1,4-galactosyltransferase, P1/Pk synthase, EC 2.4.1.228), encoded by *A4GALT* (GenBank Accession number NG_007495.2), catalyzes the transfer of galactose from the nucleotide sugar donor UDP-Gal to the terminal galactose on lactosylceramide (LacCer), initiating the globo-series pathway by forming globotriaosylceramide (Gb3, P^k^ antigen, CD77, Galα1→4Galβ1→4Glc-Cer) [3]. The same enzyme synthesizes the P1 antigen (nLc5, Galα1→4Galβ1→4GlcNAcβ1→3Galβ1→4Glc-Cer) from paragloboside (nLc4), which belongs to the neolacto-series of glycosphingolipids (GSLs). We showed before that Gb3/CD77 synthase with p.Q211E substitution acts sequentially with Gb4 synthase producing NOR1 and NOR2, which differ in the number of Galα1→4GalNAcβ1 units [4], the presence of which underlies the rare NOR blood group phenotype. This structure was never before described in mammals but found in the frog *Rana ridibunda* [5,6], and its molecular function is unknown. Thus, Gb3/CD77 synthase p.Q211E produces all three human P1PK blood group antigens: two with terminal Galα1→4Galβ1 disaccharide (Gb3 and P1) and one antigen terminating with Galα1→4GalNAcβ1 (NOR). Recently, it was shown that Gb3/CD77 synthase can also add galactose to Galβ1-Cer, creating galabiosylceramide (Galα1→4Galβ1-Cer) [7]. Gb3 is a receptor for pathogens and toxins, such as uropathogenic strains of *Escherichia coli*, and Shiga toxins (Stxs), which are released by Shiga toxin-producing *E. coli* (STEC) and *Shigella dysenteriae* of serotype 1. These toxins present a serious threat to the human population because they may cause haemorrhagic colitis and often fatal hemolytic uremic syndrome (HUS) [8,9]. Worldwide, the number of STEC infections is estimated at 2.8 million annually [10,11]. The mechanism of Stx cytotoxicity is quite well understood, but some aspects of receptor recognition remain unclear. There is a general agreement that the main receptor for Stx is Gb3, while the hypothesis that glycoproteins containing Galα1→4Gal could be alternative receptors was long abandoned but recently revisited [12,13,14].

In most vertebrates the Galα1→4Gal moiety is primarily expressed on glycosphingolipids. In contrast, birds belonging to the *Neoaves* parvclass (but not to *Galloanserae* parvclass and *Paleognathae* infraclass) express Galα1→4Gal on glycoproteins, including egg white glycoproteins [15]. In a recently published article, Morimoto et al. [16] reported two different genes encoding the Gb3/CD77 synthase homologs in the wild pigeon (*Columba livia*). They proposed that one of them, named *A4GALT*1, encodes an enzyme that synthesizes GSLs but not glycoproteins. However, the authors did not provide the sequence of *A4GALT*1, nor did they evaluate the activity of its product. The enzyme encoded by the other gene, called *A4GALT*2, was analyzed in detail and it showed dual activity; its main acceptors are glycoproteins, but it can also synthesize Gb3, although at a lower rate. In addition, the authors showed that glycoproteins with terminal Galα1→4Gal may act as functional receptors for Shiga toxins, although much less effective than glycosphingolipids [16].

In this study, we attempted to shed more light on these discrepancies by evaluating the specificities of two different pigeon Gb3/CD77 synthases encoded by two paralogous genes, one of which we serendipitously discovered while conducting this study.

## 2. Results

When genomic DNA from *C. livia* was used to amplify the avian *A4GALT* gene using primers described by Suzuki et al. [17], we found that two distinct DNA fragments were amplified. One of them was identified as avian Gb3/CD77 synthase previously described in [17] (GenBank Accession Number: NM_001315524.1). The other was its paralog with 93% nucleotide identity (Supplementary Figure S1). We named the former Gb3/CD77 synthase and its paralog P and M, respectively (hereinafter referred to as P and M enzymes). To evaluate specificities of P and M enzymes, both genes were cloned into pCAG vector and used to transfect human teratocarcinoma 2102Ep cell line, which is a good model for studying GSL-synthesizing enzymes because the cells are rich in neutral GSLs [6,18].

**Figure 1:**
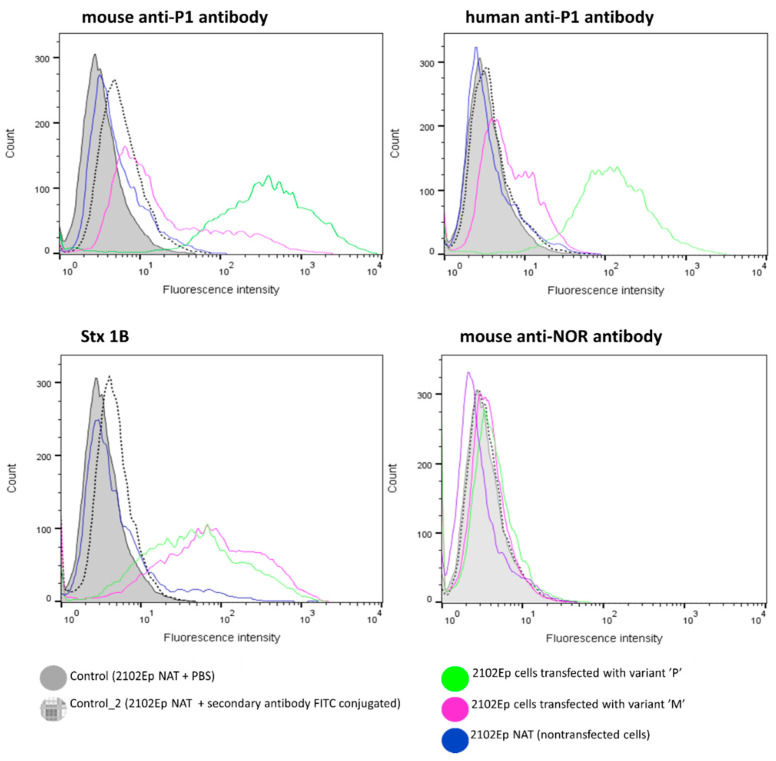
Flow cytofluorometry analysis of 2102Ep cells transfected with the vectors encoding M and P enzymes using mouse anti-P1 antibody (specificity: P1 and Gb3), human anti-P1 antibody (specificity: P1), mouse anti-NOR antibody and Stx1B; NAT: nontransfected 2102Ep cells used as a negative control.

Flow cytofluorometry analysis with mouse and human anti-P1 antibodies showed that cells transfected with the vector encoding P had markedly increased levels of Galα1→4Gal expression compared to cells transfected with the vector encoding M (Figure 1). Some anti-P1 reactivity was also detectable in nontransfected 2102Ep cells because they express endogenous (human) Gb3/CD77 synthase that produces small amounts of the P1PK antigens in those cells [6]. Cells transfected with vectors encoding M or P bound similar quantities of Shiga toxin subunit 1B (Stx1B). Nontransfected cells showed a similar Stx1B binding pattern to those of anti-P1 antibodies.

**Figure 2:**
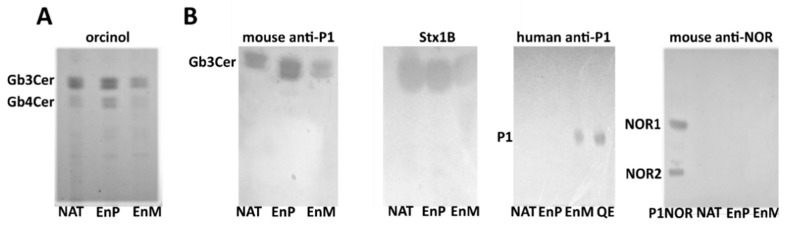
HPTLC and immuno-overlays of neutral glycosphingolipids isolated from 2102Ep cells. (**A**) orcinol staining; (**B**) immuno-overlays of mouse anti-P1, human anti-P1 and anti-NOR (nor118) antibodies and Stx1B. NAT: nontransfected 2102Ep cells, EnM: 2102Ep cells transfected with the vector encoding enzyme M; EnP: 2102Ep cells transfected with the vector encoding enzyme P; P1NOR: neutral GSLs isolated from human RBCs with P_1_NOR phenotype, QE: neutral GSLs isolated from 2102Ep cells transfected with vector encoding human Gb3/CD77 synthase with p.Q211E substitution (as a positive control for the anti-NOR antibody). Positions of bands representing individual GSLs are shown on the left.

In the HPTLC analysis of GSLs from nontransfected 2102Ep cells and the cells transfected with the M or P variant (‘the M enzyme’ or ‘the P enzyme’ cells), orcinol staining detected two forms of Gb3Cer and Gb4Cer (with hydroxy and nonhydroxy fatty acids in the ceramide moieties), which were the major neutral GSLs (Figure 2A). The mouse anti-P1 antibody detected a band corresponding to Gb3Cer, while human anti-P1 antibody detected the P1 antigen, but only in the neutral GSL fraction prepared from the M-expressing cells (Figure 2B). No binding of anti-NOR antibody to any GSL samples was observed (Figure 2B). Shiga toxin subunit 1B detected only Gb3Cer in all samples, generating the same binding patterns as the mouse anti-P1 antibody.

**Figure 3:**
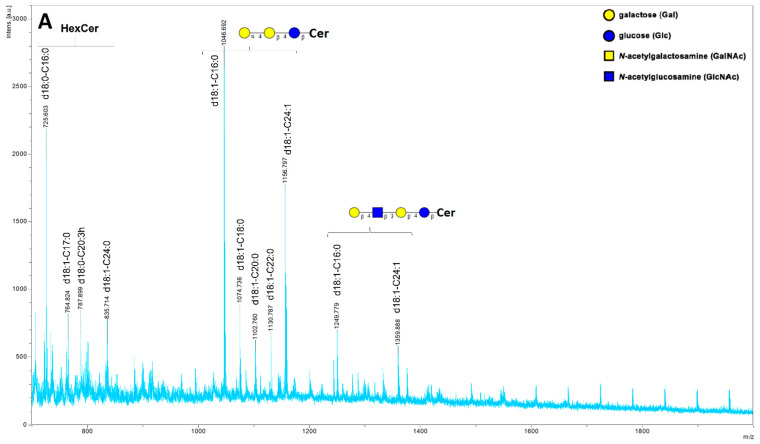

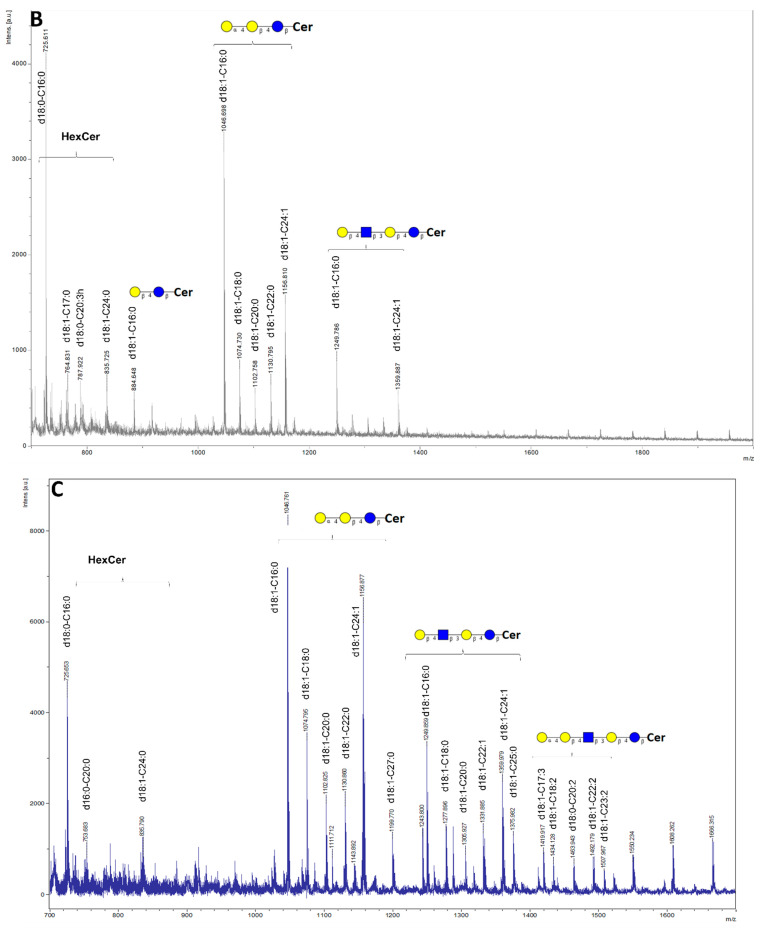
MALDI–TOF mass spectra of GSLs isolated from 2102Ep cells nontransfected or transfected with vectors encoding the M or P enzyme. (**A**) nontransfected 2102Ep cells; (**B**) 2102Ep cells transfected with vector encoding enzyme P; (**C**) 2102Ep cells transfected with vector encoding enzyme M. Individual peaks are annotated with predicted GSL structures (including the fatty acid chain length and saturation). The list of sugar symbols is shown in (**A**).

MALDI–TOF mass spectrometry analysis revealed that the major components of neutral GSLs isolated from nontransfected cells were: GlcCer, Gb3Cer and Gb4Cer with dominant ions at *m*/*z* 725.6 (GlcCer), *m*/*z* 1046.7 and *m*/*z* 1156.8 (Gb3Cer), *m*/*z* 1249.8 and 1359.9 for Gb4Cer, respectively. GSLs from ‘the P enzyme’ cells contained similar ions to GSLs from nontransfected 2102Ep cells, with dominant ions at *m*/*z* 725.6 (GlcCer), *m*/*z* 1046.7 (Gb3Cer) and *m*/*z* 1249.8 (Gb4Cer). Ion for LacCer was also identified at *m*/*z* 884.6 (Figure 3B). The M-expressing cells contained the same ions: GlcCer, Gb3Cer and Gb4Cer. Moreover, we also found ions in the range from *m*/*z* 1419.9 (C17:3 ceramide isoform) to 1507.96 (C23:2 ceramide isoform) that may correspond to the human P1/NOR antigen (Figure 3C). Since anti-NOR antibody did not react with any GSLs in HPTLC immune-assays, we assume that these ions were derived from the P1 antigen. For almost every glycosphingolipid, we observed three to five peaks corresponding to isoforms with different fatty chain length. Among ions identified for the M enzyme, especially for the P1 glycosphingolipid, we observed many rare and unusual fatty acid lengths and modifications (multiple unsaturated hydrocarbon chains).

**Figure 4:**
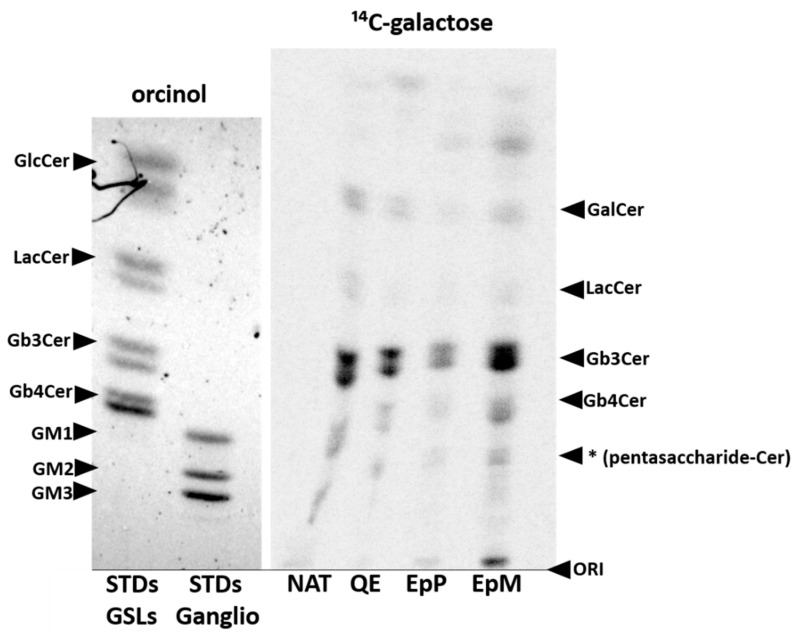
HPTLC analysis of metabolically labeled (^14^C-galactose) glycosphingolipids isolated from nontransfected 2102Ep cells and cells transfected with vectors encoding the M and P enzymes. STDs GSLs: standards for neutral fraction of glycosphingolipids (two bands from hydroxy and nonhydroxy form of each GSL); STDs Ganglio: standards for gangliosides; NAT: nontransfected 2102Ep cells; QE: 2102Ep cells transfected with the vector encoding human Gb3/CD77 synthase (p.Q211E variant); EnP: 2102Ep cells transfected with the vector encoding enzyme P; EnM: 2102Ep cells transfected with the vector encoding enzyme M. The asterisk indicates an unidentified glycosphingolipid.

Additionally, we evaluated glycosphingolipids in 2102Ep cells transfected with vectors encoding human or avian Gb3/CD77 synthases (the M or P enzyme) using metabolic labelling with ^14^C-galactose (Figure 4). We found that in all examined clones (nontransfected 2102Ep cells, and the cells transfected with vectors encoding human Gb3/CD77 synthase, or the avian P and M enzymes) the major GSL was Gb3Cer, with additional bands representing GalCer, LacCer, Gb4Cer and unidentified pentasaccharide glycosphingolipid. All clones revealed similar pattern of GSLs, except for the P-expressing cells, in which a slightly lower amount of GSLs was detected. In addition, GSLs derived from P- and M-expressing cells contained fast migrating bands.

**Figure 5:**
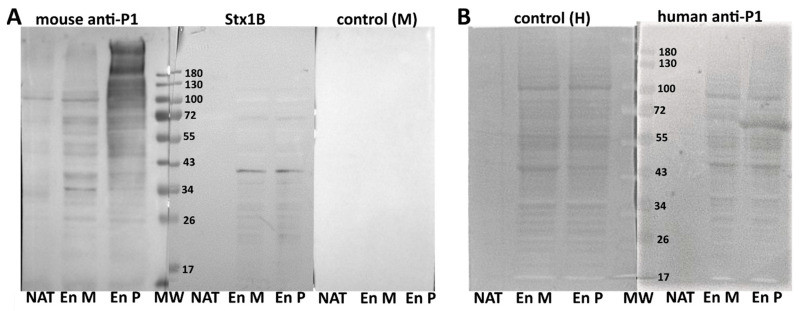
Western blotting of 2102Ep transfected with vectors encoding the M and P enzymes: (**A**) mouse anti-P1 antibody and Stx1B binding to protein lysates isolated from 2102Ep cells transfected with the genes encoding M and P enzymes. Control (M): secondary antibody only. (**B**) Human anti-P1 antibody binding to protein lysates isolated from 2102Ep cells transfected with the M and P genes with a negative control without primary antibody (control (H). MW (kDa): molecular weight standard Page Ruler PreStained (Thermo Fisher Scientific, Waltham, MA, USA); NAT: nontransfected 2102Ep cells; EnP: 2102Ep cells transfected with the vector encoding enzyme P; EnM: 2102Ep cells transfected with the vector encoding enzyme M.

Western blotting showed that binding of mouse anti-P1 antibody to the lysates prepared from the P-expressing cells was much stronger than to the lysates from the M variant-transfected cells, especially to the bands representing proteins with molecular weight of 100 kDa and higher. Binding of human anti-P1 antibody to the lysates obtained from both kinds of cells was similar and seemed to be mostly nonspecific. Interestingly, in lysates from cells transfected with vectors encoding M and P enzymes, Stx1B bound mainly to a band with MW of 40 kDa (Figure 5).

**Figure 6:**
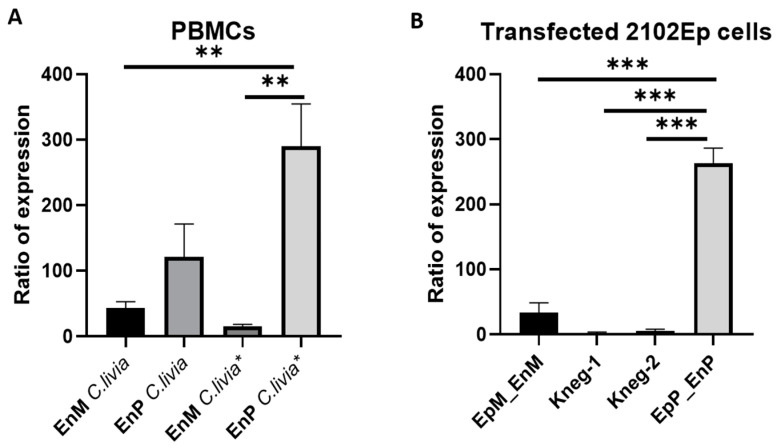
Quantitative analysis of the M and P gene transcripts. (**A**) PBMC (peripheral blood mononuclear cells); EnM: the gene encoding the M enzyme, EnP: the gene encoding the P enzyme; *C. livia*: racing pigeon, *C. livia* *: fancy pigeon; (**B**) NAT: nontransfected 2102Ep cells; EpM_EnM: 2102Ep cells transfected with the vector encoding the M enzyme, EpP_EnP: 2102Ep cells transfected with the vector encoding the P enzyme; Kneg-1: 2102Ep cells transfected with the gene encoding the M enzyme with a probe for the gene encoding the P enzyme; Kneg-2: 2102Ep cells transfected with the gene encoding the P enzyme with a probe for the gene encoding the M enzyme. Relative expression data were evaluated using one-way ANOVA test with Bonferroni post hoc test; **/***—statistical significance (**—*p* < 0.001; ***—*p* < 0.0001).

To rule out the presence of both investigated genes in one clone (which could lead to misinterpretation of enzyme specificities), we performed quantitative analyses of the transcript levels of genes encoding Gb3/CD77 synthases M and P in 2102Ep cells. The relative transcript levels were approximately 270 and 25 for the P and M enzymes, respectively (Figure 6). There was no nonspecific binding of probes.

## 3. Discussion

Human Gb3/CD77 synthase is an unusual glycosyltransferase because it can add a galactose residue to two different GSL acceptors, creating Gb3 (P^k^) and the P1 antigen. Moreover, the p.Q211E substitution broadens the acceptor specificity even further, making it able to also transfer galactose to Gb4, creating the NOR antigen, and altogether produce four or even five different GSLs [7,19]. In contrast, its homolog from *C. livia*, which has been the only nonhuman Gb3/CD77 synthase evaluated so far, can attach galactose mostly to glycoproteins, while its activity towards GSLs is much lower [16,17]. In addition, an active enzyme was found only in neoavian species of birds whereas birds belonging to the *Galloanserae* parvclass and *Paleognathae* infraclassis do not express Galα1→4Gal structures [15,20,21]. Biosynthesis of terminal Galα1→4Gal moiety in birds takes place by the action of Gb3/CD77 synthase, which is encoded by the *A4GALT* gene in humans. Suzuki et al. and Morimoto et al. [16,17] described two *C. livia* homologs of human *A4GALT* and called them *A4GALT*1 and *A4GALT*2 (the latter seems to encode the enzyme we call ’P‘ in this study). While *A4GALT*1 appeared to be GSL-specific, *A4GALT*2, which the authors evaluated in detail, synthesized Galα1→4Gal termini on glycoproteins and, to a lesser extent, on glycosphingolipids [16]. In this study, using the same primers as described by Suzuki et al. [17] and *C. livia* genomic DNA as the template, we cloned and isolated two genes with 93% nucleotide homology. One is the previously described *A4GALT*2 and we named its product ’Gb3/CD77 synthase P‘ (the P enzyme), while the other we designated ’Gb3/CD77 synthase M’ (the M enzyme). We do not know if M is the previously described *A4GALT*1 because the authors did not provide its nucleotide sequence. The qPCR analysis of mRNA from fancy pigeon showed that both genes are expressed in peripheral blood mononuclear cells (PBMC), and the level of P gene transcript is 20 times greater than the level of M transcript. Thus, the protein level of Gb3/CD77 synthase P is likely also greater than the level of M.

To evaluate the acceptor specificities of Gb3/CD77 synthases M and P, we transfected cells of the human 2102Ep teratocarcinoma line (which express large amounts of neutral glycosphingolipids) with vectors encoding M or P. The cells were evaluated using flow cytofluorometry, HPTLC, mass spectrometry, metabolic labeling and Western blotting. In flow cytofluorometry, we observed weak binding of both anti-P1 antibodies (mouse and human) to the cells transfected with vector encoding the M enzyme and markedly higher binding of these antibodies to P-expressing cells. On the other hand, the binding of Stx1B to both kinds of cells was similar. Despite the presence of G at position 631 of the M open reading frame (which broadens the acceptor specificity of the human Gb3/CD77 synthase and underlies the NOR phenotype) and availability of the NOR1 precursor (Gb4) in 2102Ep cells, we did not detect any binding of the anti-NOR antibody. This suggests that the ability of Gb3/CD77 synthase to produce the NOR antigen depends on additional, perhaps structural, factors [6]. HPTLC immuno-overlays of neutral GSLs isolated from the 2102Ep cells revealed that the major component recognized by mouse anti-P1 antibody in both transfected and nontransfected cells was Gb3, and this pattern was corroborated by the Stx1B HPTLC overlay assays. However, the human anti-P1 antibody reacted only with GSLs isolated from M- but not P-expressing cells. This suggests that only the M enzyme is able to produce the P1 antigen in 2102Ep cells. MALDI–TOF analysis of neutral GSLs showed that nontransfected 2102Ep cells and P-expressing cells had similar compositions of GSLs, including those that commonly occur in human cells such as GlcCer/GalCer, Gb3Cer or Gb4Cer. In contrast, GSLs from M-expressing cells contained additional peaks likely representing the P1 antigen. Interestingly, the spectra also included ions derived from GSLs containing unusual fatty acid chains, which require further analysis. These data suggest that the M but not P enzyme can synthesize extended GSLs. To cross-check these results, we employed ^14^C-galactose labelling of the 2102Ep cells transfected with vectors encoding P or M, using the same method as Morimoto et al. [16] but with additional removal of acidic GSLs. We did not find significant differences in GSL composition between control (nontransfected), human and avian *A4GALT*-transfected cells. The P-expressing cells contained less neutral GSLs in general. Extra fast-migrating bands likely represented galactosyl-alkylacyl and galactosyl-diacyl glycerols because it was previously shown that hydroxy and nonhydroxy fatty acid-containing Cer and diglycerides could be galactosylated by galactosyltransferases depending on their local availability [22] (Figure 4). Due to the endogenous expression of *A4GALT*, the 2102Ep cell line may not be ideal for metabolic labelling experiments, but it was useful for addressing the question of whether the M enzyme can synthesize the NOR antigen, which we found it cannot. This is intriguing because the M enzyme contains E at the same position as the variant of human Gb3/CD77 synthase with p.Q211E substitution, which underlies its NOR synthase activity. The inability of M to synthesize the NOR GSLs despite having the substitution that is causal in NOR-positive humans brings an interesting new perspective to the ongoing mechanistic studies on the Gb3/CD77 synthase activity changes.

Western blotting of 2102Ep cell lysates revealed that both P- and M-expressing cells produced Galα1→4Gal moiety on glycoproteins, but the P-expressing cells produced significantly more than the M-expressing cells. This may be caused by the differences in transcript levels because the P gene expression was approximately five times higher than that of M. On the other hand, Stx1B recognized similar levels of glycoproteins in P and M-expressing cells, with no significant differences in binding specificity or strength. Thus, both enzymes (M and P) can synthesize glycoproteins. Taken together, our results show that Gb3/CD77 synthase P can synthesize Galα1→4Gal termini mostly on glycoproteins, and, to a markedly lesser extent, on GSLs. Thus, we postulate that the P paralog of avian Gb3/CD77 synthase is a mostly glycoprotein-specific enzyme.

Data on the P enzyme shed more light on the results reported by Morimoto et al., who used metabolic labelling to evaluate its specificity because, in addition, we show that the P enzyme produces markedly less GSLs than M [16]. This stands in stark contrast to an earlier study describing pigeon Gb3/CD77 synthase (presumably encoded by *A4GALT*2) expressed in HEK293T cells [17], which did not detect Gb3 in flow cytofluorometry with mouse anti-P1 and anti-Gb3 antibodies. The results of our in-depth analysis presented here buttress the observations by Morimoto and coworkers, and fill a significant gap in our understanding of how birds synthesize Galα1→4Gal-decorated glycans.

In contrast to the P enzyme, the Gb3/CD77 synthase M, which we possibly describe here for the first time, can transfer Gal onto two kinds of acceptors: glycoproteins and glycosphingolipids, although its activity is generally lower in comparison to P. Similar to the P enzyme, we found the gene encoding M in every analyzed individual from *Columbidae* clade.

Interestingly, Tian and coworkers recently showed that the activity of human Gb/CD77 synthase depends on a poorly characterized protein called lysosomal-associated protein transmembrane 4 alpha (LAPTM4A) [23,24]. Our *in silico* analysis suggests that the pigeon enzymes are significantly less dependent on interactions with LAPTM4A (data not shown). In addition, the human Gb3/CD77 synthase has shown similar activity in nonhuman cells, including canine [25]. This suggests that different Gb3/CD77 synthases may productively partner with xenogenic LAMPT4A, and thus the nonavian biological context of our transfection experiments did not significantly affect our findings.

In summary, we characterized two highly paralogous avian genes that encode two enzymes with Gb3/CD77 synthase activity. One of them, which we named P, was previously described [13,18], but we found that the enzyme is primarily active towards glycoproteins and produces negligible amounts of glycosphingolipids. The other one, which we called M, can efficiently synthesize Galα1→4Gal moieties on both glycoproteins and glycosphingolipids. We believe that both enzymes produce Shiga toxin receptors in the avian cells. Moreover, we cannot rule out other players. As it has already been shown by bioinformatic analysis, birds might have up to seven different genes encoding Gb3/CD77 synthase and/or Gb3/CD77 synthase-like enzymes (supplementary material in [26]). The range of acceptor specificities and roles that they play remain to be explored. 

The sensitivity of cells expressing the Galα1→ 4Gal-capped glycoconjugates to Shiga toxins is important for our understanding of the relationship between diarrheagenic *E. coli* strains found in birds and their hosts, which is still a matter of debate. It is generally accepted that the presence of diarrheagenic strains in some species may represent a public health risk. Shiga toxin-producing (STEC) and enteropathogenic *E. coli* (EPEC) represent two of at least six pathotypes of human diarrheagenic *E. coli* that colonize avian guts and might be considered zoonotic pathogens. However, the avian mechanism of resistance to toxins is unknown [27]. It has been shown that birds can harbor STEC, but, like cattle, they seem refractory to Stx and could be a spillover host. Starlings and pigeons would be likely culprits in a spillover scenario because these two avian species can inhabit city buildings, parks and playgrounds, where humans may be exposed to these animals and their feces [28,29,30]. Wild birds were first identified as a potential source of STEC infection in 1997 [31]. Since then, STEC has been isolated from pigeons, sparrows, starlings and other avian species [32]. Studies have shown that starlings carrying STEC O157 can spread bacteria at levels greater than 100 CFU/g of feces for as long as 13 days post-colonization [33]. In addition, birds migrate long distances, so they could facilitate the transmission of STEC over large areas. Transmission between bird populations may also contribute to the spread of STEC [28]. Birds can contaminate produce when they defecate on farms. STEC carriers have also been reported in poultry. The prevalence of STEC O157:H7 in chickens ranged from 0 to 1.5%, while in turkeys up to 7.5% of fecal samples tested positive [34].

Recent reports proposed that human Gb3/CD77 synthase can also attach Gal residues to glycoprotein acceptors, contrary to the long-standing belief that it can only use glycosphingolipids [14,35]. If the human and avian enzymes indeed synthesize similar products, it would rule out the possibility that birds are refractory to Shiga toxins due to a lack of functional receptors. Intriguingly, *Neoaves* seem to nullify Stx invasion despite producing at least two active forms of Gb3/CD77 synthase, which synthesize both types of receptors (glycoproteins and glycosphingolipids). Deciphering this phenomenon will likely require looking beyond the toxin-receptor recognition aspect of STEC infection. All published articles thus far that describe avian Gb3/CD77 synthase, including ours, show that birds, in general, produce more than one Gb3/CD77 synthase. Specificity of the vast majority of those enzymes is unknown and teasing out potential differences between them could help us understand the avian paradox of Shiga toxin resistance.

## 4. Materials and Methods

All of the antibodies used in experiments described in this paper are listed in Supplementary Table S1. MS Office software and Inkscape [36] tools were used for data visualization.

### 4.1. Isolation of Genomic DNA from Pigeon Red Blood Cells, Amplification and Cloning

Genomic DNA was isolated from livers of sacrificed pigeons using Quick Blood DNA Purification Kit (EURx, Gdansk, Poland) according to the protocol provided by the supplier. Primers and conditions used in the PCR reaction for amplification of the *A4GALT* gene are listed in Supplementary Table S3A,B. PCR was performed using an MJ Mini gradient PCR apparatus (Bio-Rad, Hercules, CA, USA) in 100 µL reaction mixes containing 200 ng of pigeon genomic DNA (template), 0.2 mM dNTPs, HF Taq buffer with MgCl_2_ (20× dilution), 0.2 mM forward and reverse primers, and 1 unit of HF Taq polymerase (Thermo Fisher Scientific, USA). The PCR products were purified using Gel-Out (A&A Biotechnology, Gdansk, Poland). The PCR products were further amplified using primers with restriction sites: XhoI (F) and NotI (R) (Thermo Fisher Scientific, Waltham, MA, USA) and cloned into the pCAG vector (kindly provided by Prof. Peter W. Andrews, University of Sheffield, Sheffield, UK) [18]. To test for the presence of *A4GALT* paralogs, the PCR products were digested with PaeI and HincII (Thermo Fisher Scientific, Waltham, MA, USA); 500 ng of purified PCR products were digested in 37°C for 6 h and analyzed by electrophoresis in 1% agarose gel with MIDORI green dye (Nippon Genetics Europe, Duren, Germany).

### 4.2. Cell Culture and Transfection

The 2102Ep teratocarcinoma cells (kindly provided by Prof. Peter W. Andrews, University of Sheffield) [18] were grown in DMEM containing 4.5 g/L glucose (Gibco, Waltham, MA, USA) supplemented 10% fetal bovine serum (Gibco) and 2 mM GlutaMAX (Gibco, Waltham, MA, USA) in a 37 °C, 5% CO_2_ atmosphere. Cultures were passaged using 0.25% trypsin/1mM EDTA. For experiments, cells were resuspended in fresh medium and seeded into fresh tissue culture flasks; 24 h before transfection, cells were seeded on six-well plates (2 × 10^4^ cells per well). Monolayers were approximately 70% confluent on transfection day (day ’0‘). Four hours before transfection, the culture medium was replaced with fresh DMEM without supplements. Cells were transfected with 3 µg of plasmid DNA using polyethyleneimine (Polysciences, Warrington, PA, USA). Briefly, plasmid DNA was diluted with buffer containing 0.15 M NaCl and 20 mM HEPES pH 7.5 and mixed with 15 µg of polyethyleneimine. The transfection mixture was incubated for 30 min at room temperature and then added to each well. After 24 h, the transfection medium was replaced with fresh supplemented medium. For stable transfection assays, antibiotic selection was initiated 48 h after transfection, using 0.2–1 µg/mL concentration of puromycin (Thermo Fisher Scientific, Waltham, MA, USA). Each experiment was performed at least three times.

### 4.3. Flow Cytofluorometry

The 2102Ep cells transfected with vectors encoding pigeon Gb3/CD77 synthases were harvested using 0.25% trypsin/1 mM EDTA; 2 × 10^5^ cells/mL were washed 3 times with PBS and analyzed on FACSCalibur (BD Biosciences, Franklin Lakes, NJ, USA) using mouse monoclonal anti-P1 antibody, human monoclonal anti-P1 antibody (dilution 1:2000) or Shiga toxin subunit 1B (Stx1B, final concentration 1 µg/mL). Cells were washed (all washes were done with PBS), suspended in 100 µL of diluted primary antibody or Stx1B (diluted in PBS with 1% BSA and 0.05% NaN_3_), incubated for 45 min on ice and washed again. Stx1B flow cytofluorometry assay involved incubations with a secondary (100 µL mouse anti-6His diluted 1:100, Thermo Fisher Scientific, Waltham, MA, USA) and tertiary (100 µL FITC-labeled polyclonal rabbit anti-mouse, anti-human immunoglobulin diluted 1:100, Thermo Fisher Scientific, Waltham, MA, USA) antibody for 45 min on ice in the dark. Before analysis, cells were washed and resuspended in PBS (0.5 mL). The number of events analyzed was 10,000/gated cell population, and data analyses were carried out using Flowing Software 2.5.1 [37].

### 4.4. Protein Lysates

Protein lysates were prepared using CelLytic M^®^ reagent (Sigma, St. Louis, MO, USA) according to the manufacturer’s protocol: 100 µL of transfected and nontransfected 2102Ep cells were mixed with CelLytic M^®^ reagent containing 10 mM EDTA pH 8.0 and proteinases inhibitor cocktail (Thermo Fisher Scientific, Waltham, MA, USA) and incubated in RT for 15 min with gentle shaking. Next, the mixture was centrifuged at 14,000× *g*, 4° C for 15 min. Supernatants were collected and used for analysis. Total protein concentration in samples was measured using a Pierce™ BCA Protein Assay Kit (Thermo Fisher Scientific, Waltham, MA, USA).

### 4.5. SDS-PAGE and Western Blotting

The protein lysates were separated in the presence of SDS (Roth, Karlsruhe, Germany) using 10% polyacrylamide gel, according to the Laemmli method [38] and visualized with Coomassie Brilliant Blue R-250 (Roth, Karlsruhe, Germany) or transferred to the nitrocellulose membrane (Roth, Karlsruhe, Germany). The PageRuler Prestained Protein Ladder (Thermo Fisher Scientific, Waltham, MA, USA) was used as a protein standard. The proteins fractionated by SDS-PAGE were transferred to nitrocellulose membrane [39], overlaid with anti-P1 antibodies (diluted 1:100) or Stx1B (final concentration 1 µg/mL and anti-6xHis antibody diluted 1:1000 as secondary) and anti-mouse antibody conjugated with alkaline phosphatase (diluted 1:1000) for 45 min in room temperature and visualized using NBT/BCIP reagents (nitro blue tetrazolium/5-bromo-4-chloro-3-indolyl phosphate) (Roth, Karlsruhe, Germany) in 100 mM Tris pH 9.2 with the addition of 1 mM MgCl_2_, 1 mM MnCl_2_ and 150 mM NaCl.

### 4.6. Extraction and Purification of Glycosphingolipids

The isolation and fractionation of glycosphingolipids and the orcinol staining were performed using the standard procedures applied previously to human red blood cells [19]. Briefly, glycolipids were extracted from 2102Ep cells with chloroform/methanol, and the neutral glycolipids were separated from the phospholipids and gangliosides and desalted using Sephadex G-25 resin. The purified glycosphingolipids were solubilized in chloroform/methanol (2:1, *v*/*v*), applied to HPTLC plates (Kieselgel 60, Merck, Darmstadt, Germany) and analyzed by high-performance thin-layer chromatography (HPTLC), using chloroform/methanol/water (55:45:9, *v*/*v*/*v*) for development. The dried plates were immersed in 0.05% polyisobutylmethacrylate (Sigma-Aldrich, St. Louis, MO, USA) in hexane for 1 min, dried, rinsed with 1X TBS (0.05 M Tris, 150 mM NaCl, pH 7.4) and blocked in 5% human serum albumin (HSA) in TBS for 1 h. For antibody assays, plates were overlaid with (1) mouse anti-P1 or human anti-P1 antibody or mouse anti-NOR (clone nor118, [40]) antibody (diluted 1:100 with 1% BSA/TBS); (2) biotinylated goat anti-mouse IgG antibody or goat anti-human IgG antibody, diluted 1:1000 with 1% BSA/TBS, used in the case of anti-P1 and anti-NOR antibodies (for Stx1B assay, this step was preceded by incubation with anti-6xHis, diluted 1:1000); (3) ExtrAvidin-alkaline phosphatase conjugate diluted 1:5000 with 1% BSA/TBS/0.05% Tween20 for 1 h; and (4) the substrate NBT/BCIP solution (Sigma-Aldrich, St. Louis, MO, USA). Each HPTLC experiment was repeated at least three times. For the Stx1B (final concentration of toxin 1 µg/mL) HTPLC assay, step 2 was preceded by incubation with anti-6His antibody.

### 4.7. Mass Spectrometry Analysis of Glycosphingolipids

MALDI–TOF (matrix-assisted laser desorption/ionization–time-of-flight) mass spectrometry of glycosphingolipids was carried out on ultrafleXtreme MALDI–TOF/TOF instrument (Bruker Daltonics, Bremen, Germany). Samples were dissolved in chloroform/methanol (2:1, *v*/*v*). Norharmane (9H-pyrido[3,4-b]indole (Sigma-Aldrich, St. Louis, MO, USA) was used as a matrix (10 mg/mL, chloroform/methanol, 2:1, *v*/*v*). Spectra were scanned in the range of *m*/*z* 700–3500 in the reflectron-positive mode. External calibration was applied using the peptide calibration standard (Bruker Daltonics, Bremen, Germany).

### 4.8. Metabolic Labelling of Glycosphingolipids and TLC Analysis

Experiments with metabolic labelling of glycosphingolipids were performed as described previously [16]. Briefly, nontransfected 2102Ep cells and cells transfected with vector encoding enzyme M or P were seeded on six-well plates (3 × 10^5^ cells/well) in fully supplemented medium as described above. For metabolic labeling, ^14^C-galactose (PerkinElmer, Waltham, MA, USA) was used. Cells were incubated overnight in Opti-MEM medium supplemented with 1% of Nutridoma (Sigma-Aldrich, St. Louis, MO, USA) and 0.2 µCi of ^14^C-galactose in standard conditions. Glycosphingolipids were isolated according to the protocol from Section 4.5 of this paper with slight modification. In HPTLC analysis, chloroform/methanol/0.25% CaCl_2_ (65:35:8, *v*/*v*/*v*) was used as development solvent.

### 4.9. Quantitative Analysis of Avian A4GALT Transcripts

Total RNA from peripheral blood mononuclear cells (PBMCs) isolated from sacrificed pigeons as described before [41] were prepared using GeneMATRIX Universal RNA Purification Kit (EURx, Gdansk, Poland) and the complementary DNAs (cDNAs) were synthesized using SuperScript III First-Strand Synthesis Kit (Thermo Fisher Scientific, Waltham, MA, USA), with oligo(dT) primers. Quantitative polymerase chain reaction (qPCR) was performed on 75 ng of cDNA using the 7500 Fast Real-Time PCR System Thermo Fisher Scientific, Waltham, MA, USA), according to the manufacturer’s instruction. The *A4GALT* transcripts were detected with Custom TaqMan Gene Expression Assay (Thermo Fisher Scientific, Waltham, MA, USA). The TaqMan assay targeted differences in the sequence of genes encoding enzymes P and M, respectively. The transcript quantities were normalized to the chicken *GAPDH* endogenous control (also prepared using Custom TaqMan Gene Expression Assay, Thermo Fisher Scientific, Waltham, MA, USA). All samples were run in triplicates. Data were analyzed using Sequence Detection Version 1.3.1 software (Thermo Fisher Scientific, Waltham, MA, USA). Target nucleotide sequences and qPCR conditions are shown in Supplementary Table S3.

### 4.10. Statistical Analysis

For measurements of expression level ratios between the two pigeon *A4GALT* genes, one-way ANOVA with the Bonferroni post hoc test was used. All analyses were prepared using GraphPad Prism (version 8.0.0 for Windows, GraphPad Software, San Diego, CA, USA, www.graphpad.com, accessed on 16 September 2020).

## Data Availability

The datasets generated during and/or analyzed during the current study are available from the corresponding author on reasonable request or are included in this published article (and its Supplementary Material files).

## Supplementary Materials

The following are available online at https://www.mdpi.com/article/10.3390/ijms22189761/s1.

## Abbreviations

GSL: glycosphingolipid; Gb3: globotriaosylceramide; Stx: Shiga toxin; Stx1B: Shiga toxin 1B subunit; STEC: Shiga toxin-producing *Escherichia coli*; HUS: hemolytic-uremic syndrome; HPTLC: high-performance thin-layer chromatography; qPCR: quantitative polymerase chain reaction; MALDI–TOF: matrix-assisted laser desorption/ionization–time-of-flight; PBMC: peripheral blood mononuclear cells.

## References

[1] Lairson L.L., Henrissat B., Davies G.J., Withers S.G. (2008). Glycosyltransferases: Structures, Functions, and Mechanisms. Annu. Rev. Biochem..

[2] Breton C., Šnajdrová L., Jeanneau C., Koča J., Imberty A. (2005). Structures and mechanisms of glycosyltransferases. Glycobiology.

[3] Furukawa K., Kondo Y., Furukawa K. (2014). UDP-gal: Lactosylceramide alpha 1,4-galactosyltransferase (*A4GALT*). Handbook of Glycosyltransferases and Related Genes.

[4] Kaczmarek R., Buczkowska A., Mikołajewicz K., Krotkiewski H., Czerwinski M. (2014). P1PK, GLOB, and FORS Blood Group Systems and GLOB Collection: Biochemical and Clinical Aspects. Do We Understand It All Yet?. Transfus. Med. Rev..

[5] Mourad R., Morelle W., Neveu A., Strecker G. (2001). Diversity of O-linked glycosylation patterns between species. JBIC J. Biol. Inorg. Chem..

[6] Suchanowska A., Kaczmarek R., Duk M., Lukasiewicz J., Smolarek D., Majorczyk E., Jaskiewicz E., Laskowska A., Waśniowska K., Grodecka M. (2012). A Single Point Mutation in the Gene Encoding Gb3/CD77 Synthase Causes a Rare Inherited Polyagglutination Syndrome. J. Biol. Chem..

[7] Akiyama H., Ide M., Yamaji T., Mizutani Y., Niimi Y., Mutoh T., Kamiguchi H., Hirabayashi Y. (2021). Galabiosylceramide is present in human cerebrospinal fluid. Biochem. Biophys. Res. Commun..

[8] Johannes L., Römer W. (2009). Shiga toxins—From cell biology to biomedical applications. Nat. Rev. Genet..

[9] Bruyand M., Mariani-Kurkdjian P., Gouali M., de Valk H., King L., Le Hello S., Bonacorsi S., Loirat C. (2018). Hemolytic uremic syndrome due to Shiga toxin-producing Escherichia coli infection. Médecine et Maladies Infectieuses.

[10] Majowicz S.E., Scallan E., Jones-Bitton A., Sargeant J.M., Stapleton J., Angulo F.J., Yeung D.H., Kirk M. (2014). Global Incidence of Human Shiga Toxin–ProducingEscherichia coliInfections and Deaths: A Systematic Review and Knowledge Synthesis. Foodborne Pathog. Dis..

[11] Heredia N., García S. (2018). Animals as sources of food-borne pathogens: A review. Anim. Nutr..

[12] Okuda T., Numata S.-I., Ito M., Ohta M., Kawamura K., Wiels J., Urano T., Tajima O., Furukawa K., Furukawa K. (2006). Targeted Disruption of Gb3/CD77 Synthase Gene Resulted in the Complete Deletion of Globo-series Glycosphingolipids and Loss of Sensitivity to Verotoxins. J. Biol. Chem..

[13] Zumbrun S.D., Hanson L., Sinclair J.F., Freedy J., Melton-Celsa A.R., Rodriguez-Canales J., Hanson J.C., O’Brien A.D. (2010). Human Intestinal Tissue and Cultured Colonic Cells Contain Globotriaosylceramide Synthase mRNA and the Alternate Shiga Toxin Receptor Globotetraosylceramide. Infect. Immun..

[14] Szymczak-Kulus K., Weidler S., Bereznicka A., Mikolajczyk K., Kaczmarek R., Bednarz B., Zhang T., Urbaniak A., Olczak M., Park E.Y. (2021). Human Gb3/CD77 synthase produces P1 glycotope-capped N-glycans, which mediate Shiga toxin 1 but not Shiga toxin 2 cell entry. J. Biol. Chem..

[15] Suzuki N., Laskowski M., Lee Y.C. (2006). Tracing the history of Galα1-4Gal on glycoproteins in modern birds. Biochim. Biophys. Acta-Gen. Subj..

[16] Morimoto K., Suzuki N., Tanida I., Kakuta S., Furuta Y., Uchiyama Y., Hanada K., Suzuki Y., Yamaji T. (2020). Blood group P1 antigen–bearing glycoproteins are functional but less efficient receptors of Shiga toxin than conventional glycolipid-based receptors. J. Biol. Chem..

[17] Suzuki N., Yamamoto K. (2010). Molecular Cloning of Pigeon UDP-galactose:β-d-Galactoside α1,4-Galactosyltransferase and UDP-galactose:β-d-Galactoside β1,4-Galactosyltransferase, Two Novel Enzymes Catalyzing the Formation of Galα1–4Galβ1–4Galβ1–4GlcNAc Sequence. J. Biol. Chem..

[18] Liew C.-G., Draper J.S., Walsh J., Moore H., Andrews P.W. (2008). Transient and Stable Transgene Expression in Human Embryonic Stem Cells. Stem Cells.

[19] Kaczmarek R., Szymczak-Kulus K., Bereznicka A., Mikołajczyk K., Duk M., Majorczyk E., Krop-Watorek A., Klausa E., Skowrońska J., Michalewska B. (2018). Single nucleotide polymorphisms in *A4GALT* spur extra products of the human Gb3/CD77 synthase and underlie the P1PK blood group system. PLoS ONE.

[20] Suzuki N., Laskowski M., Lee Y.C. (2004). Phylogenetic expression of Galα1-4Gal on avian glycoproteins: Glycan differentiation inscribed in the early history of modern birds. Proc. Natl. Acad. Sci. USA.

[21] Suzuki N., Nawa D., Su T.-H., Lin C.-W., Khoo K.-H., Yamamoto K. (2013). Distribution of the Galβ1-4Gal Epitope among Birds: Species-Specific Loss of the Glycan Structure in Chicken and Its Relatives. PLoS ONE.

[22] Nagai K.-I., Takahashi N., Niimura Y. (2016). Novel biosynthesis of monogalactosyl-alkylacyl glycerolipid in Mop8 fibroblast cells transfected with a ceramide galactosyltransferase gene. Biomed. Res. Clin. Pr..

[23] Tian S., Muneeruddin K., Choi M.Y., Tao L., Bhuiyan R.H., Ohmi Y., Furukawa K., Furukawa K., Boland S., Shaffer S.A. (2018). Genome-wide CRISPR screens for Shiga toxins and ricin reveal Golgi proteins critical for glycosylation. PLoS Biol..

[24] Pacheco A.R., Lazarus J., Sit B., Schmieder S., Lencer W., Blondel C.J., Doench J.G., Davis B.M., Waldor M.K. (2018). CRISPR Screen Reveals that EHEC’s T3SS and Shiga Toxin Rely on Shared Host Factors for Infection. mBio.

[25] Müller S.K., Wilhelm I., Schubert T., Zittlau K., Imberty A., Madl J., Eierhoff T., Thuenauer R., Römer W. (2016). Gb3-binding lectins as potential carriers for transcellular drug delivery. Expert Opin. Drug Deliv..

[26] Shapiro M.D., Kronenberg Z., Li C., Domyan E.T., Pan H., Campbell M., Tan H., Huff C.D., Hu H., Vickrey A.I. (2013). Genomic Diversity and Evolution of the Head Crest in the Rock Pigeon. Science.

[27] Sanches L.A., Gomes M.D.S., Teixeira R.H.F., Cunha M.P.V., De Oliveira M.G.X., Vieira M.A.M., Gomes T., Knobl T. (2017). Captive wild birds as reservoirs of enteropathogenic E. coli (EPEC) and Shiga-toxin producing E. coli (STEC). Braz. J. Microbiol..

[28] Persad A.K., LeJeune J.T. (2014). Animal Reservoirs of Shiga Toxin-Producing Escherichia coli. Microbiol. Spectr..

[29] Borges C.A., Maluta R.P., Beraldo L.G., Cardozo M.V., Guastalli E.A., Kariyawasam S., DebRoy C., Ávila F.A. (2017). Captive and free-living urban pigeons ( Columba livia ) from Brazil as carriers of multidrug-resistant pathogenic *Escherichia* coli. Veter- J..

[30] Fadel H.M., Afifi R., Al-Qabili D.M. (2017). Characterization and zoonotic impact of Shiga toxin producing Escherichia coli in some wild bird species. Veter- World.

[31] Wallace J.S., Cheasty T., Jones K. (1997). Isolation of Vero cytotoxin-producing Escherichia coli O157 from wild birds. J. Appl. Microbiol..

[32] Pedersen K., Clark L. (2007). A review of Shiga toxin Escherichia coli and Salmonella enterica in cattle and free-ranging birds: Potential association and epidemiological links. Human–Wildlife Conflicts.

[33] Kauffman M.D., Lejeune J. (2011). European Starlings (Sturnus vulgaris) challenged with Escherichia coli O157 can carry and transmit the human pathogen to cattle. Lett. Appl. Microbiol..

[34] Doane C.A., Pangloli P., Richards H.A., Mount J.R., Golden D.A., Draughon F.A. (2007). Occurrence of Escherichia coli O157:H7 in Diverse Farm Environments. J. Food Prot..

[35] Stenfelt L., Westman J.S., Hellberg Å., Olsson M.L. (2019). The P1 histo-blood group antigen is present on human red blood cell glycoproteins. Transfusion.

[36] Schöler U., Schöler U. (2014). Inkscape.

[37] Samani F.S., Moore J.K., Khosravani P., Ebrahimi M. (2013). Features of free software packages in flow cytometry: A comparison between four non-commercial software sources. Cytotechnology.

[38] Laemmli U.K. (1970). Cleavage of Structural Proteins during the Assembly of the Head of Bacteriophage T4. Nature.

[39] Towbin H., Staehelin T., Gordon J. (1979). Electrophoretic transfer of proteins from polyacrylamide gels to nitrocellulose sheets: Procedure and some applications. Proc. Natl. Acad. Sci. USA.

[40] Duk M., Lisowska E. (2006). Presence of natural anti-Galα1-4GalNAcβ1-3Gal (anti-NOR) antibodies in animal sera. Glycoconj. J..

[41] Panda S., Ravindran B. (2013). Isolation of Human PBMCs. Bio-Protocol.

