# Supplementary material for: Two Paralogous Gb3/CD77 Synthases in Birds Show Different Preferences for Their Glycoprotein and Glycosphingolipid Substrates
Source: Int J Mol Sci. 2021 Sep 9;22(18):9761. doi: 10.3390/ijms22189761 (PMC8466213; doi:10.3390/ijms22189761)
Supplement: Supplementary file 1 [file ijms-22-09761-s001.zip › ijms-1360479-supplementary.pdf]

Supplementary Material

Two Paralogous Gb3/CD77 Synthases in Birds Show Different Preferences for Their Glycoprotein and Glycosphingolipid Substrates

Anna Bereznicka [1], Krzysztof Mikolajczyk [1], Katarzyna Szymczak-Kulus [1], Katarzyna Kapczynska [2], Edyta Majorczyk [3], Anna Modlinska [4], Tomasz Piasecki [4], Radoslaw Kaczmarek [1] and Marcin Czerwinski [1,*]

[1] Laboratory of Glycobiology, Department of Immunochemistry, Hirszfeld Institute of Immunology and Experimental Therapy, Polish Academy of Sciences, 53-114 Wroclaw, Poland; anna.bereznicka@hirszfeld.pl (A.B.); krzysztof.mikolajczyk@hirszfeld.pl (K.M.); katarzyna.szymczak-kulus@hirszfeld.pl (K.S.-K.); radoslaw.kaczmarek@hirszfeld.pl (R.K.)

[2] Laboratory of Medical Microbiology, Department of Immunology of Infectious Diseases, Hirszfeld Institute of Immunology and Experimental Therapy, Polish Academy of Sciences, 53-114 Wroclaw, Poland; katarzyna.kapczynska@hirszfeld.pl

[3] Faculty of Physical Education and Physiotherapy, Opole University of Technology, 45-758 Opole, Poland; E.Majorczyk@po.edu.pl

[4] Department of Epizootiology and Clinic of Birds and Exotic Animals, Wroclaw University of Environmental Sciences, 50-366 Wroclaw, Poland; ana.modlinska@gmail.com (A.M.); tomasz.piasecki@upwr.edu.pl (T.P.)

* Correspondence: marcin.czerwinski@hirszfeld.pl

Table S1. Antibodies used in the experiments.

Antibody	Company
**Mouse anti-P1 (clone 650, recognizes Gb3 and P1 antigen)**	Ce-Immundiagnostika (Ger-many)
**Human anti-P1 (clone P3NIL100, recognizes P1 antigen)**	Immucor Inc. (USA)
**6xHis-Tag Monoclonal Antibody (HIS.H8)**	Thermo Fisher Scientific (USA)
**Biotinylated goat anti-mouse IgG/A/M (H/L)**	Thermo Fischer Scientific(USA)
**Goat anti-Mouse IgG (H+L) Secondary Antibody, AP**	Thermo Fisher Scientific (USA)
**Biotinylated goat anti-human polyvalent immunoglobulins**	Sigma-Aldrich (MERCK) (USA)
**Alkaline phosphatase-ExtrAvidin**	Sigma-Aldrich (MERCK) (USA)

Table S2. A. Primers used for amplification and sequencing of avian *A4GALT*; abbreviations: F: for-ward primer, R: reverse primer.

Name	Sequence
**P_*A4GALT*_XhoI_F**	5′ CTCGAGATGTCCAGCTACCTGCAA 3′
**P_*A4GALT*_NotI_R**	5′ GGCGGCCGCTCACATTACAGGCCTTGAC3′

Table S2. B. PCR conditions.

Step	Temperature [°C]	Time [hh:mm:ss]	
**1. Initial denaturation**	98	00:00:30	
**2. Denaturation**	98	00:00:20	30X
**3. Annealing**	55-60	00:00:30	30X
**4. Extension/elongation**	72	00:00:20	30X
**5. Final elongation**	72	00:10:00	

Table S3. qPCR conditions, primers and probes used for quantitative analysis of avian *A4GALT* transcripts. Abbreviations: M: enzyme M, P: enzyme P.

Step	Number of Cycles	Temperature [°C]	Time [mm:ss]
**Initial denaturation**	1	95	10:00
**Denaturation**	40	95	00:15
**Annealing/Extension**		60	01:00
***q*PCR system**	7500 Fast		
**Reaction type**	96-well plate		
**Reaction volume [μL]**	20		
**Forward primer**	5′ TGTACTGAAGAACTTGAAGAA C	3′	
**Reverse primer (P)**	5′ ATGAACTCATGCTTCGG 3′		
**Reverse primer (M)**	5′ ATGAACTCATGCTTGGG 3′		
**Probe (enzyme P)**	5′ TGCCCTCGGGCTCCAGTCTC 3′		
**Probe (enzyme M)**	5′ TGCCCTTGGTATTGAGTCTC 3′		

```
P       ATGTCCAGCTACCTGCAAAAACTGACCACAGTGCTGCCGAGCCACAGGCCTGGGGCTCTG        60
M       ATGTCCAGCTACCTGCAAAAACTGACCACAGTGCTGCCGAGCCACAGGCCTGGGGCTCTG        60
        ************************************************************

P       TTTATCCTCATCATTTCATTTCTGCTCGTTGCCTCTGTTGTGTTCTACCAGAGAACTGGG        120
M       TTTATCCTCATCATTTCATTTCTGCTCGTTGCCTCTGTTGTGTTCTACCAGAGAACTGGG        120
        ************************************************************

P       AAGGACACTGAAGGCCAGCTCTACCACTCGCCTACACAGAACAGGTCTGAAGACTTTTTG        180
M       AAGGACGCTGAGGGCCAGCTCTATGGCTTGTCTATACAAAACAGGTGTAAACAGTTTTTC        180
        ****** **** ***********   ** * *** *** ******* * ** * ***** 

P       ACTTCTCCTCCCCACACCATTGCTGGTGGGTCCCCTCCTTCCCCAGGGGATGTGTTTTTT        240
M       GCTTCCCCTCCCCACACCATTGCTGGTGGGCCTCCTCCTTCCCCAGGGGATGTGTTTTTT        240
         **** ************************ * ***************************

P       GTGGAGACCTCTGAGCGAATTAAACCAAGTTACCTGTTCACGTGCTCTGTGGAGTCAGCG        300
M       GTGGAGACCTCTGAGCAAACTAACCCAAGTTACCTGTTCACGTGGTCTGTGGAGTCAGCG        300
        **************** ** *** ******************** ***************

P       GCCCGGGCACACCCTGGAACACGGGTTGTGGTGCTCATGAAAGGCCTGGCAAAGGGGAAT        360
M       GCCCGGGCACACCCTGGAACACGGGTTGTGGTGCTCATGAAAGGCCTGGCAAAGGGGAAT        360
        ************************************************************

P       GTCTCATTGCCCAGCCACTGGGCATTCTCATTGCTGAGCCGCTTCCCCAACGTGGAGATC        420
M       GTCTCATTGCCCAGCCACTGGGCATTCTCATTGCTGAGCTGCTTCCCCAACATGGAGATC        420
        *************************************** *********** ********

P       CAGCCCCTGGACTTGGCAGAGCTTTTCTCAGGAACACCTCTGGCAAAGTGGTACTCACAG        480
M       CGGCCCCTGGACTTGGCAGAGCTTTTCTCAGGAACACCTCTGGCAAAGTGGTACTCACAG        480
        * **********************************************************

P       CCTGAGCACCAGAAGGAACCTTATTTCTTTCCCGTCCTGTCTGACGCCTGCAGAATTACC        540
M       CCTGAGCACCAGAAGGAACCTTATTTCTTTCCCGTCCTGTCTGACGCCTGCAGAATTGCC        540
        ********************************************************* **

P       ATCATGTGGAAATTTGGTGGCATCTACCTGGACACAGACTTCATTGTACTGAAGAACTTG        600
M       ATCATGTGGAAATTTGGTGGCATCTACCTGGACACAGACTTCATTGTACTGAAGAACTTG        600
        ************************************************************

P       AAGAACCTCACCAATGCCCTCGGGCTCCAGTCTCAGGATGTACTGAATGGGGCCTTCTTG        660
M       AAGAACCTCACCAATGCCCTTGGTATTGAGTCTCAGGATGTGCTGAATGGGGCCTTTCTG        660
        ******************** **  *  ************* **************  **

P       TCTTTCAAACCGAAGCATGAGTTCATGGAACTTTGCATTCAGGACTTTGTGGACAACTAC        720
M       TCCTTCAAACCCAAGCATGAGTTCATAGAACTTTGCATGCAGGACTTTGTCGATAACTAC        720
        ** ******** ************** *********** *********** ** ******

P       AATGGGTGGATCTGGGCACACCAGGGCCCAGAACTGTTAACACGTGTCTTCAAAAAGTTA        780
M       AAAAGCTGGATCTGGGGGCACCAGGGCCCACAGCTACTAACACGTGTCTTCAAGAAGTGG        780
        **  * **********  ************ * **  **************** ****  

P       TGCTCCATCAGTAATATCCAGAATGGTATGATCTGCAAAGGAGTGAGTGCTCTTCCTCCT        840
M       TGCTCTATCAGTAATATCCAAAGTGGTATGATCTGCAAAGGAGTGAGTGCTCTTCCCCCT        840
        ***** ************** * ********************************* ***

P       GATGCTCTTTATCCCATTCCATGGCAGGACTGGAAGAAATTATTTGAAGCAATCAGCTCC        900
M       GATGCTCTTTATCCCATTCCATGGCAGGACTGGAAGAAATTATTTGAAGCAATCAGCTCC        900
        ************************************************************

P       TCAGAGCTTCACAATCTCCTTAAGAACACCTATGCGGTGCACGTATGGAACAAACTGAGC        960
M       TCAGAGCTTCACAATCTCCTTAAGAACACGTATGCGGTGCATGTATGGAACAAACTGAGC        960
        ***************************** *********** ******************

P       CACGATGCAAGGCTAGAGATCACGTCCCAGGCTTTGCTGGCTCAGCTGTATTCTCAGTTC        1020
M       CACGATGCAAGGCTAGAGATCACGTCCCAGGCTTTGCTGGCTCAGCTGTATTCTCAGTTC        1020
        ************************************************************

P       TGCCCTGCCACATCTGCACAGATGAAGAAGGACTTTGAAGAGCAGTCAAGGCCTGTAATG        1080
M       TGCCCTGCCACATCTGCACAGATGAAGAAGGACTTGAAGAGCAGTCAGCGAACTGTAATG        1080
        ***********************************  *           *  ********

P       TGA          1083
M       TGA          1083
        ***
```

Figure S1. Comparison of the nucleotide sequences of the *A4GALT* paralogs from the wild pigeon (*C. livia*). Sequences were aligned with ClustalW [1]. 'P': sequence deposited in GenBank (NM_001315524.1), 'M': sequence found in our laboratory.

References

1. McWilliam, H.; Li, W.; Uludag, M.; Squizzato, S.; Park, Y.M.; Buso, N.; Cowley, A.P.; Lopez, R. Analysis Tool Web Services from the EMBL-EBI. *Nucleic Acids Res*. **2013**, 41, 597–600, doi:10.1093/nar/gkt376.
